# A Review of the Application of Seal Whiskers in Vortex-Induced Vibration Suppression and Bionic Sensor Research

**DOI:** 10.3390/mi16080870

**Published:** 2025-07-28

**Authors:** Jinying Zhang, Zhongwei Gao, Jiacheng Wang, Yexiaotong Zhang, Jialin Chen, Ruiheng Zhang, Jiaxing Yang

**Affiliations:** 1 Beijing Key Laboratory for Precision Optoelectronic Measurement Instrument and Technology, School of Optics and Photonics, Beijing Institute of Technology, Beijing 100081, China; 3220230637@bit.edu.cn (Z.G.); 3120225346@bit.edu.cn (J.W.); 3120220661@bit.edu.cn (Y.Z.); 3220230635@bit.edu.cn (J.C.); 3220235077@bit.edu.cn (R.Z.); 3120220654@bit.edu.cn (J.Y.); 2 Yangtze Delta Region Academy, Beijing Institute of Technology, Jiaxing 314001, China; 3 State Key Laboratory of Chips and Systems for Advanced Light Field Display, School of Optics and Photonics, Beijing Institute of Technology, Beijing 100081, China

**Keywords:** seal vibrissae structure, vortex-induced vibrations, fluid mechanics, bionics

## Abstract

Harbor seals (*Phoca vitulina*) have excellent perception of water disturbances and can still sense targets as far as 180 m away, even when they lose their vision and hearing. This exceptional capability is attributed to the undulating structure of its vibrissae. These specialized whiskers not only effectively suppress vortex-induced vibrations (VIVs) during locomotion but also amplify the vortex street signals generated by the wake of a target, thereby enhancing the signal-to-noise ratio (SNR). In recent years, researchers in fluid mechanics, bionics, and sensory biology have focused on analyzing the hydrodynamic characteristics of seal vibrissae. Based on bionic principles, various underwater biomimetic seal whisker sensors have been developed that mimic this unique geometry. This review comprehensively discusses research on the hydrodynamic properties of seal whiskers, the construction of three-dimensional geometric models, the theoretical foundations of fluid–structure interactions, the advantages and engineering applications of seal whisker structures in suppressing VIVs, and the design of sensors inspired by bionic principles.

## 1. Introduction

Through long-term evolution, animals and plants have developed perception systems that are highly adaptable to complex environments. Among them, the visual system—shared by humans and most animals—serves as a fundamental modality for locating external targets. For pinnipeds, however, the need for vision to operate across two vastly different media—air and water—poses significant evolutionary challenges [1]. In contrast, direct sensory modalities such as olfaction and mechanoreception, characterized by their compactness and high signal-to-noise ratio (SNR), have attracted increasing attention from both biologists and engineers [2]. Notable examples include the lateral line systems in fish and Xenopus frogs [3], as well as the whiskers of pinnipeds such as seals [4], which amplify subtle mechanical disturbances from the environment through structural specialization to achieve refined perception. In recent years, in-depth investigations into these biological sensing systems have inspired the development of bioinspired sensors—particularly, seal whiskers, whose undulating morphology has shown remarkable performance in suppressing vortex-induced vibrations (VIVs), thereby garnering considerable research interest.

Among the many biological mechanoreceptors with the characteristics of “small size and high signal-to-noise ratio”, the whiskers of pinnipeds have become a hot research object due to their special three-dimensional geometric structure and strong ability to suppress self-excited vibrations. Pinnipeds are typically classified into three phylogenetic groups: phocids (true seals), otariids (sea lions), and odobenids (walruses) [5,6]. A shared trait among them is their streamlined body shape, which significantly reduces hydrodynamic drag and enhances underwater swimming efficiency. Early studies hypothesized that pinnipeds might employ sonar-like echolocation mechanisms similar to those of odontocetes (e.g., dolphins and sperm whales) during prey tracking [7]. However, subsequent research over several decades has confirmed that pinnipeds, limited by their auditory system’s frequency range, have not evolved the capability to generate or receive high-frequency sound signals.

Instead, pinnipeds—like otters [8] and Australian water rats [9]—possess a high density of follicle–sinus complexes (FSCs) around the muzzle. These FSCs contain mechanosensory neurons capable of converting minute mechanical stimuli—captured by whiskers—into neural signals, a process analogous to the electroacoustic transduction in sonar systems [10]. As a result, pinnipeds can use their densely arrayed vibrissae to track prey with high precision, earning them the reputation of “experts” in underwater passive hydrodynamic sensing [4,10,11,12,13]. Notably, the characteristic wavy structure of seal whiskers plays a crucial role in disrupting vortex shedding (i.e., Kármán vortex streets), thereby reducing background noise and enhancing SNR—an essential factor in prey localization during hunting [14,15,16].

To explore how pinnipeds detect and localize hydrodynamic trails using their vibrissae, researchers have conducted studies on species such as the harbor seal (*Phoca vitulina*) [17], California sea lion (*Zalophus californianus*) [17], and Pacific walrus (*Odobenus rosmarus divergens*) [18]. Findings show that pinnipeds, especially harbor seals, possess distinct undulated 3D whisker geometries capable of sensing vortex wakes left by moving objects. Moreover, aspects such as whisker distribution, asymmetric movement patterns, and large vibration amplitudes have been identified as critical for prey localization in sea lions [19]. Microscopic analyses reveal that whisker surfaces are not smooth but exhibit surface protrusions, often resembling sinusoidal waves. These structures have been experimentally proven to suppress VIVs and enhance hydrodynamic sensing performance in noisy aquatic environments. In summary, a deeper understanding of the mechanical structure and sensing mechanisms of seal whiskers holds significant promise for the development of next-generation bioinspired flow sensors, with applications in underwater surveillance, target detection, and marine resource exploration.

**Figure 1:**
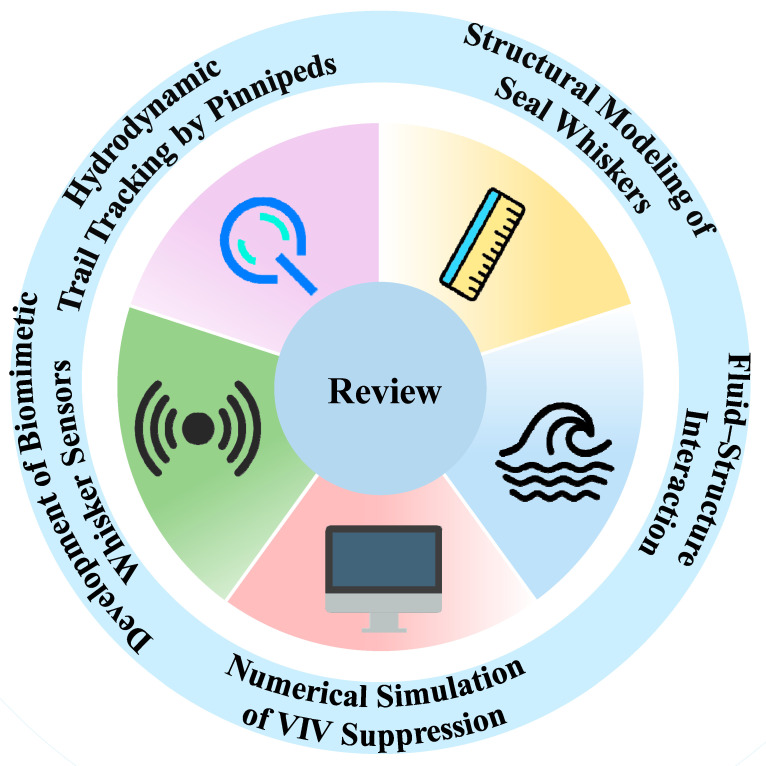
Overview framework of the review.

This review provides a comprehensive overview of advances in the study of seal whisker-inspired vibration suppression mechanisms and sensor development (see Figure 1). The discussion is organized into several parts: observations of hydrodynamic trail tracking by whiskers; modeling of whisker morphology; fluid–structure interaction analysis; and recent progress in numerical simulations and biomimetic sensor design, offering insights for future marine sensing technologies.

## 2. Investigation of Seal Hydrodynamic Characteristics and the Development of a Whisker Structural Model

### 2.1. Progress in Seal Hydrodynamic Characteristics Research

In dark, turbid seawater, spotted seals are capable of tracking fish that swam by up to 30 s earlier, even though they lack the active acoustic tracking abilities found in other odontocetes (such as dolphins and whales) [7]. Although seals possess an amphibious visual system that functions both on land and underwater—adjusting adaptively through changes in corneal thickness and pupillary spacing [1]—this system becomes ineffective in the low-visibility conditions of deep ocean waters; yet, their predatory performance remains unimpaired. Many researchers believe that the dense whisker structures near the nasal sinuses play a crucial role in this phenomenon, hypothesizing that seals can detect the hydrodynamic trails left by prey without relying on vision. This remarkable ability, a result of natural selection, represents a unique adaptation not found in other animals and has thus attracted significant scientific interest in the sensory mechanisms of seals. To underscore the pivotal role of whiskers in prey tracking, researchers have initiated studies focusing on the hydrodynamic characteristics of prey as detected by seals [20].

In 1979, researchers from Dalhousie University, led by Renouf [21], proposed that seals utilize their whiskers to assist in sensing complex underwater environments and localizing prey. Renouf and colleagues controlled two variables—water turbidity and whether the whiskers were clipped—to investigate whether spotted seals could conduct normal foraging activities under these conditions, while also analyzing the role of whiskers in the process. The experiment involved four groups of spotted seals hunting for trout, and they found that without the aid of whiskers, the seals were unable to capture trout in a short period. Although the experimental conditions and conclusions at the time were insufficient to definitively prove that whiskers play a critical role in the foraging process, this study nonetheless sparked significant scientific interest in the function and structure of seal whiskers. This discovery opened a new realm in seal whisker research, thereby paving the way for innovative studies in fields such as bionics and biology.

**Figure 2:**
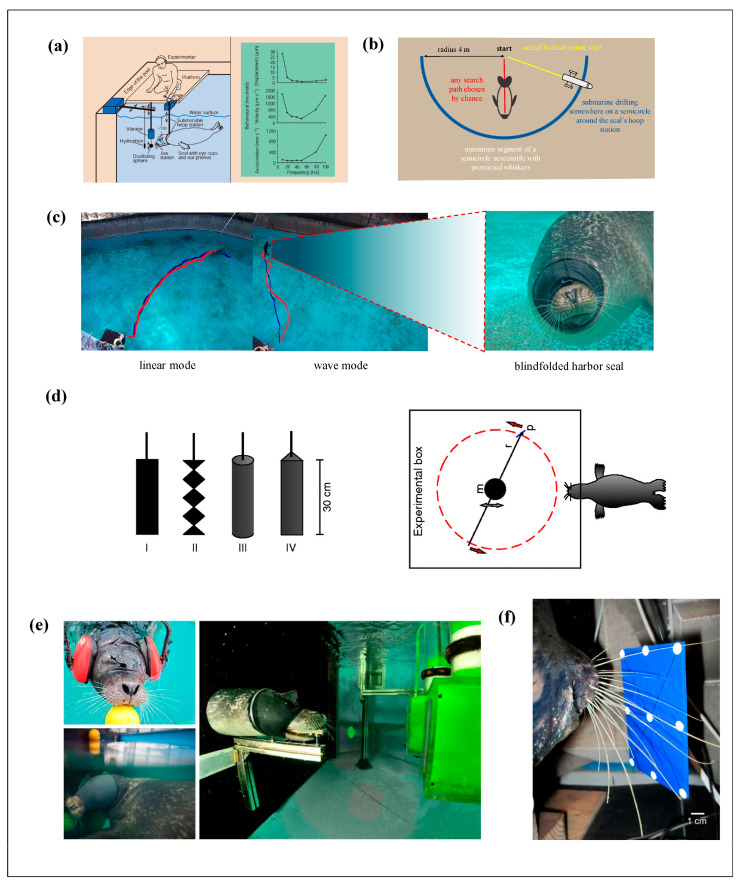
Experimental study on the hydrodynamic characteristics of seals. (**a**) Experimental device for tracking a vibrating ball by a harbor seal. (left) Displacement, velocity, and acceleration thresholds of a harbor seal under stimulation [32]. (**b**) Schematic diagram of a harbor seal tracking a submarine trajectory, adapted from [22]. (**c**) Two tracking modes of harbor seals, linear mode and wave mode; the red line represents the actual trajectory of the seal tracking, and the blue line represents the hydrodynamic trajectory [24]. (**d**) Schematic diagram of vortex generators of different shapes (left) and experimental device [25,26]. (**e**) Experiment of blindfolded seals tracking vortex rings [29]. (**f**) Experimental test device [30].

Dehnhardt et al. [4] were the first to reveal the ability of seal whiskers to serve as underwater sensory organs, detecting subtle hydrodynamic disturbances. The research team employed a vibrating sphere capable of generating different frequencies, controlling both the vibration frequency (10–100 Hz) and the distance between the sphere and the seal’s lower jaw (i.e., the whiskers, 5–50 cm), to simulate the effects of varying intensities of minor underwater water flow disturbances on seal foraging. To eliminate interference from ambient acoustic and optical signals, the seals’ eyes and ears were covered. The experimental results demonstrated that when the stimulus signal exceeded 50 Hz, the seals exhibited significant responses in both acceleration and velocity (as illustrated in Figure 2a). This indicates that, even in the absence of visual and auditory cues, seals can utilize their whiskers to sense external water flow stimuli.

Considering that the water flow changes induced by the vibrating sphere were extremely subtle—far weaker than the hydrodynamic trails generated by swimming fish—Dehnhardt et al. [22] further hypothesized that seal whiskers function as sensory organs for foraging, enabling seals to locate prey by tracking the hydrodynamic trails left behind by passing fish. To test this hypothesis, they designed an experiment where the seals’ eyes and ears were covered and spotted seals tracked the hydrodynamic trail left by a miniature submarine, as shown schematically in Figure 2b. The experimental protocol involved first generating a stable water flow trail with the miniature submarine, then turning off the submarine’s engine to observe the seals’ tracking performance. Previous studies have shown that the hydrodynamic wake produced by swimming fish persists much longer than the duration of the fish’s passage [23]. Twenty seconds after the submarine’s engine was shut off, the water flow velocity was approximately 16 mm/s, which is comparable to the flow fluctuations induced by a 30 cm goldfish swimming by. Although this flow velocity is very low, it was still noticeably higher than the environmental background noise and surpassed the sensitivity threshold of seal whiskers [4]. The experiment confirmed that seals can indeed detect subtle water flow fluctuations via their whiskers, enabling them to track targets generating such weak water movements. In the absence of whiskers, seals would be unable to effectively detect hydrodynamic trails and, consequently, would be impaired in their foraging activities.

Schulte-Pelkum et al. [24] addressed the shortcomings in the experimental design of Dehnhardt et al. [22] by employing spotted seals as trail generators instead of using a miniature submarine, thereby more accurately replicating the hydrodynamic trails produced by fish and other marine organisms. Their study demonstrated that the seal whisker system is optimally sensitive to frequencies between 10 and 100 Hz, with peak sensitivity at 50 Hz, which corresponds well with the hydrodynamic signals generated by fish. Using Particle Image Velocimetry (PIV) to visualize the hydrodynamic trails, they observed that seals exhibited two distinct tracking patterns (Figure 2c): a linear pattern, occurring in approximately 64% (278 cases) of the experiments, and a fluctuating pattern, occurring in approximately 34% (150 cases) of the experiments. In either mode, seals were ultimately able to follow the precise trail. Since the duration of actual fish trails is longer than that of the artificially generated trails produced by the miniature submarine, the experimental results of Schulte-Pelkum et al. are more convincing.

Furthermore, Wieskotten et al. [25,26] employed PIV technology for the first time to reveal that seals can determine an object’s direction, size, and shape based on the reverse vortices present in the object’s wake (Figure 2d). Niesterok et al. [27,28] demonstrated that seal whiskers are sensitive to the jets produced by the respiration of benthic fish (Figure 2e). Prior to these studies, many investigations were unable to clarify which information within the hydrodynamic trail spotted seals use to extract directional cues; these studies revealed that spotted seals rely on the “vortex street” signals embedded in the hydrodynamic trail for foraging. Krueger et al. [29] examined the ability of spotted seals to detect and analyze the direction of individual vortices (Figure 2f) and found that seals can perceive the smallest angular deviation of a single vortex to be 5.7°. Murphy et al. [30] applied vibrational stimuli to seals, confirming that they are most sensitive to frequencies between 20 and 250 Hz, with optimal sensitivity at around 80 Hz—this frequency range is related to hydrodynamic stimulation; however, the key variables and mechanisms underlying their perception require further investigation. Seals detect the hydrodynamic trails produced when fish swim by, and these trails consist of complex vortex structures that vary according to the fish’s body shape and swimming style. Notably, variations in the age and whisker structure among different spotted seals, as well as differences in the size and shape of the vortex streets generated by various fish, mean that the sensitive frequency range of seal whiskers is not entirely consistent across studies.

In addition to research on the hydrodynamic characteristics of seals, scientists have also investigated the mechanical properties of spotted seal whiskers. Hans et al. [31] designed two experimental setups to measure various mechanical properties of the whiskers, specifically determining the elastic modulus, damping, and natural frequency. The measurements indicate that the elastic modulus decreases along the length of the whisker, while the damping remains constant along its length.

In summary, the whiskers of spotted seals are capable of accurately tracking complex hydrodynamic trails (as shown in Table 1) and distinguishing between trail generators of different sizes and shapes [25,26]. Moreover, the whiskers can perceive the movement direction of complex hydrodynamic trails [4] and detect threshold flow velocities within the flow field. Further studies have revealed that the detection threshold for hydrodynamic motion in spotted seals is 0.25 mm/s [17].

### 2.2. Establishment of the Structural Model of Seal Whiskers

Scientific investigations into the hydrodynamic properties of seals have highlighted the critical role of their whiskers in underwater sensing. Among pinnipeds, the morphology of vibrissae differs across phylogenetic lineages. *Otariids* (e.g., sea lions) and *odobenids* (e.g., walruses) possess relatively smooth whiskers, whereas most *phocids* (true or earless seals), such as *Phoca vitulina* (harbor seal), exhibit undulated or wavy whisker structures. These undulations are believed to enhance the signal-to-noise ratio (SNR) of underwater flow perception during swimming [17].

**Figure 3:**
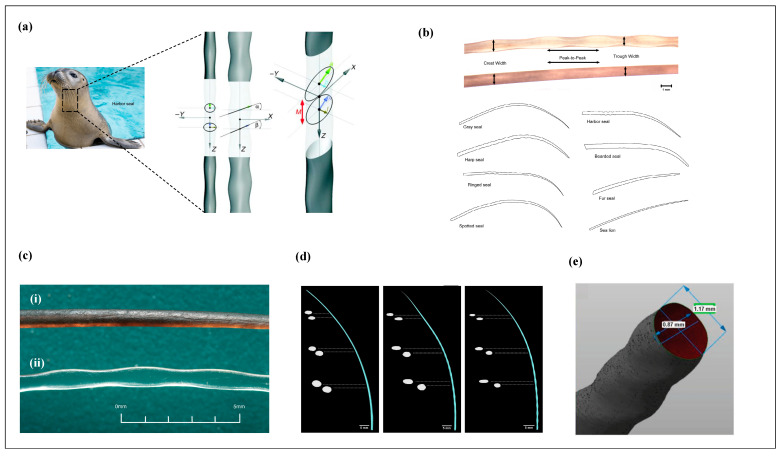
The morphological structure of seal whiskers. (**a**) Seven model parameters of the morphological structure of harbor seal and harbor seal whiskers [17]. (**b**) Two-dimensional morphological structure images of eight pinnipeds [35]. (**c**) Surface structure of smooth whiskers and wavy whiskers [36]; (**i**) Smooth whiskers (California sea lion) and (**ii**) wavy whiskers (harbor seal) surface structure. (**d**) CT data scan reconstruction images of three pinnipeds, from left to right: California sea lion, elephant seal, harbor seal [36]. (**e**) Whisker structure constructed by blue-light scanning [38].

Although both harbor seals and California sea lions can use their whiskers for hydrodynamic trail-following, their whisker morphologies are markedly distinct (Figure 3a). Seal whiskers typically feature a beaded or serrated shape, while sea lion whiskers are smoother with less prominent surface undulations. Mechanical testing and computational fluid dynamics (CFD) simulations have demonstrated that harbor seals suppress vortex-induced vibrations (VIVs) by an order of magnitude more effectively than sea lions [17].

Harbor seals are capable of detecting and tracking the wake of prey that passed by up to 30 s earlier. This exceptional ability is attributed to the unique wavy structure of their whiskers, which possess an elliptical cross-section and are oriented at a specific angle relative to the central axis (Figure 3a). Microscopic observations have revealed that along the longitudinal direction, seal whiskers follow a sinusoidal waveform [17,33], characterized by periodic crests and troughs. This morphological pattern effectively minimizes self-generated hydrodynamic noise during swimming and amplifies flow signals induced by the movement of prey.

Hanke et al. [17] were the first to quantitatively describe the structural morphology of seal whiskers, introducing a seven-parameter model to characterize their undulated geometry (Figure 3a). Using high-resolution micro-cameras, they captured detailed images of harbor seal vibrissae and defined seven parameters corresponding to the whisker’s sinusoidal structure: four elliptical radii (a, b, k, l), two inclination angles (α, β), and a half-period (M).

Specifically, parameters a and b represent the semi-major and semi-minor axes of the elliptical cross-section at the crest, measuring 0.595 mm and 0.24 mm, respectively. Similarly, k and l correspond to the semi-major and semi-minor axes at the trough, measuring 0.475 mm and 0.29 mm, respectively. The crests and troughs appear periodically along the length of the whisker, with inclination angles of 15.27° and 17.6° relative to the plane perpendicular to the whisker’s central axis. The full spatial period of the waveform is defined as 2M.

Since the introduction of this model, numerous researchers have adopted and extended it to investigate vibrissal morphology across different pinniped species. Ginter et al. [34] conducted a statistical analysis of whiskers from harp seals (*Pagophilus groenlandicus*), hooded seals (*Cystophora cristata*), and grey seals (*Halichoerus grypus*), examining characteristics such as total whisker length, number of crests and troughs, and inter-peak spacing. They found significant interspecies differences and strong positive correlations between whisker length and the number of waveform cycles (correlation coefficients r = 0.91, 0.93, and 0.85, respectively). The authors hypothesized that the protrusions along the whisker length may serve three non-exclusive functions: drag reduction, sensitivity enhancement, and signal interference mitigation.

In a subsequent study, Ginter et al. [35] combined geometric morphometrics with traditional morphometric approaches to analyze whiskers from 92 specimens across 11 pinniped species. Using high-definition cameras, they obtained scaled images of the vibrissae for comparative analysis (Figure 3b). Their results showed that harbor seals exhibited the most prominent undulated structures, while sea lions lacked noticeable waviness. In addition, they observed that whisker lengths ranged between 60 mm and 110 mm, with considerable individual variability among specimens.

Previous analyses of seal whisker morphology primarily relied on two-dimensional representations, such as those obtained from photographic imaging. However, with advancements in technology, researchers have increasingly adopted more sophisticated techniques to investigate the microstructural features of seal vibrissae. For instance, compared to traditional optical microscopy, computed tomography (CT) scanning offers significantly clearer and more detailed cross-sectional images, enabling a more comprehensive understanding of the whiskers’ three-dimensional architecture.

Murphy et al. [36] employed high-resolution computed tomography (CT) to scan and reconstruct the whiskers of harbor seals (*Phoca vitulina*), northern elephant seals (*Mirounga angustirostris*), and California sea lions (*Zalophus californianus*), as shown in Figure 3d. Rinehart et al. [37] further conducted CT scans on 27 whiskers from harbor seals and performed statistical analyses on the extracted structural data. Their findings indicated that the parameters a, b, k, l, and M closely matched the seven-parameter model previously proposed by Hanke. However, the inclination angles α and β exhibited considerable inter-individual variability, though their distribution followed a near-Gaussian pattern within the range of −5° to 5°, a novel observation not reported in earlier studies.

In addition, Kamat et al. [38] utilized blue-light scanning technology to perform high-resolution 3D reconstructions of whiskers from both harbor seals and grey seals (*Halichoerus grypus*), as illustrated in Figure 3e. This method provided precise geometric profiles and further enriched the morphological understanding of pinniped vibrissae in three dimensions.

Table 2 summarizes the geometric models of seal whiskers established by Hanke, Ginter, Murphy, and Rinehart. The results indicate that the measurements reported by Ginter, Murphy, and Rinehart are largely consistent with one another, with overall deviations falling within a single standard deviation. It is important to note, however, that the model proposed by Hanke et al. represents an idealized simplification of whisker geometry, as it neglects both tapering and curvature along the whisker shaft.

Recent studies [36,37] have demonstrated that whisker taper and curvature vary significantly along the length of the vibrissae. From base to tip, both the semi-major and semi-minor axes decrease gradually, with the minor axis changing by approximately 0.05 mm and the major axis by around 0.1 mm. Additionally, the eccentricity of the whisker increases with distance from the base, with crest regions exhibiting more elliptical cross-sections and troughs appearing more circular. Rinehart et al. [37] further noted that harbor seal whiskers tend to be more elliptical compared to those of elephant seals. In summary, the seven-parameter model proposed by Hanke et al. is most applicable to the midsection of the whisker, whereas it fails to fully capture the geometric variations present at the proximal and distal ends.

Additional studies have shown that pinniped vibrissae are capable of detecting low-frequency hydrodynamic disturbances, regardless of whether their surface structure is undulated or smooth. In fact, at certain frequencies, smooth-surfaced whiskers—such as those of the California sea lion—have demonstrated superior sensing accuracy compared to the wavy whiskers of harbor seals. This finding suggests that surface morphology alone may not be the sole determinant of hydrodynamic performance [36]. Nevertheless, the discovery and analysis of wavy whisker structures, particularly in harbor seals, have underscored their unique advantages for underwater sensing applications. These insights have not only confirmed the irreplaceable functional benefits of undulated whisker geometries in the design of underwater sensors but have also significantly advanced the fields of sensory biology and biomimetics.

## 3. Fluid–Structure Interaction (FSI) Theory

### 3.1. Generation and Shedding of Kármán Vortex Streets

As described in Section 2, harbor seals are capable of tracking the hydrodynamic trails left by prey. More precisely, this ability relies on their vibrissae sensing vortex street signals within the wake flow. Therefore, a thorough understanding of the generation and shedding mechanisms of vortex streets is essential. This chapter focuses on the theoretical analysis of the formation and shedding of Kármán vortex streets, as well as studies related to vortex-induced vibrations (VIVs) resulting from fluid–structure interactions between the flow field and seal whiskers.

When an object is placed in a fluid flow, alternating vortices with opposite rotation directions are periodically shed from its downstream side [39]. This phenomenon, known as a “Kármán vortex street”, was first identified by the renowned fluid dynamicist Theodore von Kármán and is also referred to as a “von Kármán vortex street”. Kármán vortex streets are commonly generated behind bluff bodies exposed to steady flows and can induce vibrations in the object, leading to increased structural noise. If the vortex shedding frequency coincides with the natural frequency of the structure, resonance may occur, resulting in unpredictable dynamic behavior.

The formation and characteristics of Kármán vortex streets are a central topic in computational fluid dynamics (CFD). In studies examining the vortex-suppression capabilities of seal whiskers, a typical method involves introducing a cylindrical obstacle upstream to generate a controlled wake flow. The downstream interaction between the generated vortex street and the seal whisker model is then observed to evaluate how the flow-induced frequency affects the whisker’s structural response.

The Reynolds number (Re) is a dimensionless parameter that characterizes the viscosity of a fluid. It is defined as the ratio of inertial forces to viscous forces in a flow field and is determined by:

(1) $$\;\;Re=\frac{\rho VL}{\mu }$$

where *ρ* is the fluid density, *V* is the flow velocity, *L* is the characteristic length, and *μ* is the dynamic viscosity. An increase in the Reynolds number reflects a decreasing dominance of viscous forces relative to inertial forces.

At low Reynolds numbers, viscous forces dominate, and the interactions between fluid particles are sufficient to maintain an orderly, laminar flow state. As the Reynolds number increases, inertial forces begin to surpass viscous effects, and the influence of viscosity becomes confined primarily to the boundary layer of the flow. This transition signifies the onset of flow instability and eventual turbulence.

In this context, *f_V_* denotes the vortex shedding frequency of the periodic Kármán vortex street, *D* is the effective diameter in the cross-flow direction, and *U* represents the free-stream velocity. The Strouhal number (*St*) is a dimensionless parameter commonly used in fluid dynamics to describe the vortex shedding behavior behind bluff bodies [40] and is defined by:

(2) $$\;\;St=\frac{f_{V}\times D}{U}\approx 0.198\left(1-\frac{19.7}{Re}\right)$$

As shown in Equation (2), when the Reynolds number is relatively large, the Strouhal number (*St*) can be approximated as a constant, typically around 0.2 [41].Consequently, the vortex shedding frequency can be inferred from the flow velocity and the effective diameter of the upstream obstacle.

(3) $$\;\;C_{L}=\frac{{2F}_{L}}{\rho U^{2}A}$$

Here, *C*_*L*_ denotes the lift coefficient, *F*_*L*_ is the total lift force, *ρ* is the fluid density, *U* is the free-stream velocity, and *A* represents the projected area in the direction of the flow. According to Equation (3), the lift coefficient characterizes the ability of an object to generate lift. Under otherwise identical conditions, a higher lift coefficient indicates a stronger capacity for lift generation.

(4) $$\;\;C_{D}=\frac{{2F}_{D}}{\rho U^{2}A}$$

Here, *C*_*D*_ denotes the drag coefficient, *F*_*D*_ is the total drag force, *ρ* is the fluid density, *U* is the free-stream velocity, and *A* represents the projected area in the direction of the flow. According to Equation (4), the drag coefficient reflects the magnitude of resistance experienced by an object moving through a fluid. Under otherwise identical conditions, a higher drag coefficient indicates greater resistance acting on the object.

**Figure 4:**
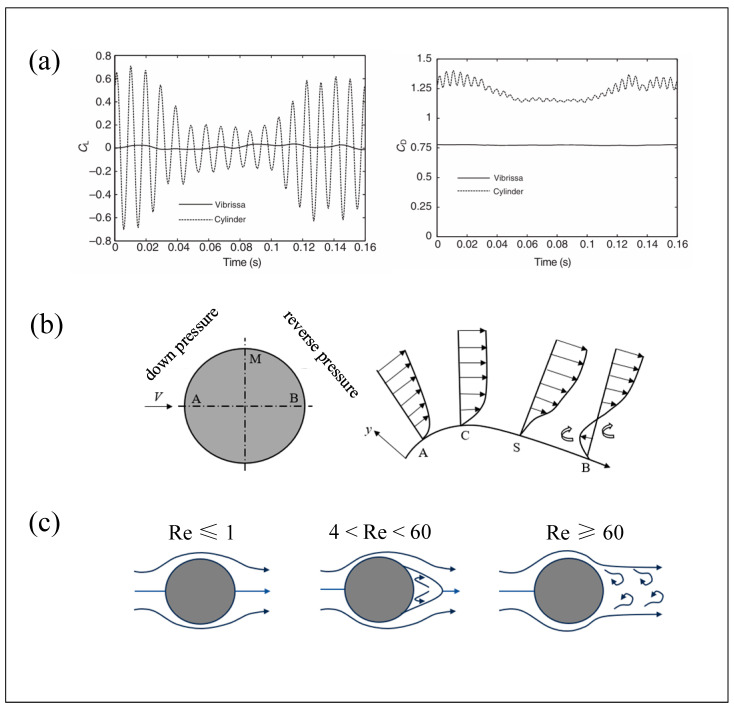
Fluid-Structure Interaction Related Theories. (**a**) Temporal variations of lift and drag coefficients. Adapted from [17]. (**b**) Vortex formation process in the wake of a circular cylinder. (**c**) Dependence of vortex shedding behavior on the Reynolds number.

At the outer edge of the boundary layer over a curved surface, both the fluid velocity and pressure vary along the flow direction. Taking the flow around a circular cylinder as an example, as illustrated in Figure 4a, a uniform incoming flow moves along the curved surface from point A to point B via point M. During the segment from A to M, the tangential velocity of the fluid gradually increases, while the pressure decreases. In this region, pressure potential energy is converted into kinetic energy. When the fluid reaches point M, its kinetic energy peaks, and pressure reaches a minimum. This region, where fluid particles are driven by a favorable pressure gradient, is referred to as the favorable pressure gradient zone.

As the flow continues from point M to point B, it enters the adverse pressure gradient zone. Here, kinetic energy begins to convert back into pressure potential energy. However, due to the inherent viscosity of the fluid, viscous friction dissipates part of the kinetic energy. As a result of both viscous dissipation and the opposing pressure gradient, the kinetic energy of the fluid particles may be depleted before reaching point B. If the adverse pressure gradient is sufficiently strong, a reverse flow may occur near the surface of the object, as shown in segment SB. The main flow of the boundary layer then separates from the surface at point S—this is known as boundary layer separation. Such flow separation can lead to vortex formation, particularly in the vicinity of the separation point. These vortices may detach and travel downstream, forming a sequence of organized or disorganized vortex structures.

The vortex shedding behavior is closely related to the Reynolds number (Re). As shown in Figure 4b, when Re ≤ 1, the boundary layer on the cylinder surface does not separate, and the streamlines remain essentially symmetric around the cylinder. When Re > 4, the boundary layer gradually develops along the cylinder wall and separates on both sides due to the adverse pressure gradient, forming two counter-rotating symmetric recirculating vortices—known as Föppl vortices. These stationary vortices attach to the rear of the cylinder, creating a relatively stable wake region. As Re increases further and exceeds 60, the vortices become unstable. The boundary layer fully separates, and vortices begin to shed alternately from both sides of the cylinder in a periodic manner. These vortices are convected downstream, forming two staggered rows of vortices in the wake—commonly referred to as the “Kármán vortex street.” The shedding frequency of the vortex street can be determined using the Strouhal number (St).

Fish utilize their lateral line system for predation, predator evasion, and interspecies communication. Studies investigating the response of the crucian carp’s lateral line system to Kármán vortex streets in uniform flow have shown that, as the vortex shedding frequency increases, the amplitude of spectral peaks also increases. This indicates a strong correlation between the neural response of the fish’s lateral line and the vortex shedding frequency [42]. Research has demonstrated that the sensitive frequency range of the seal whisker system is between 10 and 100 Hz, which closely matches the hydrodynamic signal characteristics generated by fish movements [23,43]. Coincidentally, according to Equation (2), the vortex shedding frequency of a 1 mm diameter cylinder at a swimming speed of 0.5 m/s is approximately 95 Hz—overlapping with the sensitive frequency range of the seal’s vibrissal system. Therefore, suppression of vortex-induced vibrations (VIVs) is of critical importance.

### 3.2. Generation and Research of Vortex-Induced Vibrations

When the vortex shedding frequency approaches or becomes a rational multiple of the natural frequency of a structure, it may trigger structural vibrations—referred to as vortex-induced vibrations (VIVs). VIVs are widely regarded as a major cause of fatigue failure in mechanical and structural systems. Early investigations of VIVs primarily relied on wind tunnel experiments. However, with the advancement of fluid mechanics and computational fluid dynamics (CFD), many researchers have shifted toward numerical simulations to explore the fluid–structure interaction (FSI) mechanisms in detail [44].

**Figure 5:**
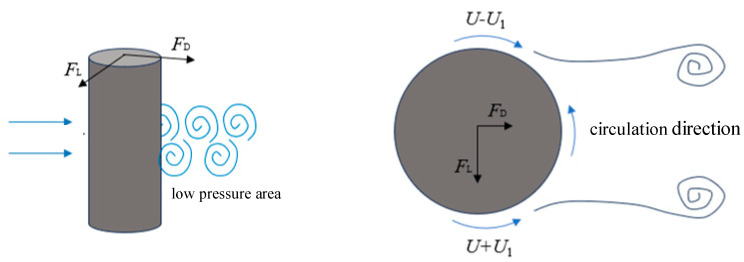
Mechanism of vortex-induced vibrations (VIVs) generation.

The periodic shedding of Kármán vortex streets exerts unsteady forces on the structure, inducing both streamwise drag (*F_D_*) and transverse lift (*F_L_*), as illustrated in Figure 5.

**Figure 6:**
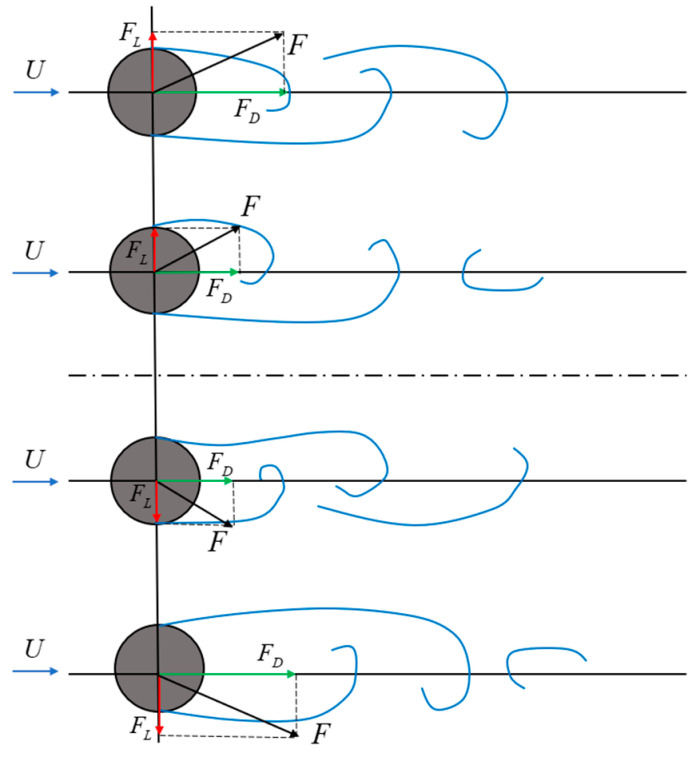
Variations of lift and drag forces over time.

When a cylinder is placed in a uniform flow with velocity *U*, alternating vortex shedding occurs around the cylinder, forming a Kármán vortex street. The shedding of vortices induces a circulation effect on the cylinder surface, characterized by a circulation velocity *U_1_*. This circulation results in a velocity difference between the upper and lower surfaces of the cylinder: the flow velocity on the upper side becomes *U* − *U_1_*, while the lower side increases to *U + U_1_*. According to potential flow theory, this velocity difference generates a pressure differential across the cylinder, which gives rise to the lift force (*F_L_*). Simultaneously, vortex shedding in the wake region creates a low-pressure zone behind the cylinder. This results in a pressure imbalance—higher pressure upstream and lower pressure downstream—which causes a net drag force (*F_D_*). The temporal evolution of the lift and drag forces is illustrated in Figure 6.

Due to the alternating vortex shedding on both sides of the cylinder, the magnitude and direction of the lift force vary sinusoidally, whereas the drag force—resulting from vortices shedding only downstream—undergoes sinusoidal variation in magnitude without a change in direction.

Over the past decades, vortex-induced vibrations (VIVs) have been extensively studied. Williamson et al. [45,46,47] conducted pioneering experiments on cross-flow VIVs of rigid cylinders with low mass ratios and two degrees of freedom. Their work identified the 2T mode and summarized four fundamental vortex shedding modes: the 2S mode (two single vortices shed per cycle, characteristic of classical Kármán vortex streets), the 2P mode (two pairs of vortices per cycle), the 2C mode (a pair of co-rotating vortices formed each half-cycle), and the 2T mode (three vortices per half-cycle, including two counter-rotating vortex pairs and a third strong vortex generated during the acceleration phase). More complex vortex patterns can be considered as combinations of these four basic modes.

Wang et al. [48,49] conducted a comprehensive review on vortex-induced vibrations (VIVs) of circular cylinders. They proposed a high-resolution numerical method based on two-dimensional simulations, employing a total variation diminishing (TVD) finite volume scheme to solve the unsteady Reynolds-averaged Navier–Stokes (URANS) equations for simulating two-dimensional riser VIVs.

Zhao et al. [50] extended the study of VIVs from two-dimensional (2D) to three-dimensional (3D) flow fields. Although many prior studies focused on amplitude and frequency responses of the cylinder, the transition of flow structures from 2D to 3D remained less explored. They investigated the vibration responses, vortex shedding modes, and flow characteristics of the cylinder under various Reynolds numbers and reduced velocities, Ur = U/(*f*_nw_*·D*), where *f*_nw_ denotes the natural frequency of the system. Results showed that at low Reynolds numbers, the 2D and 3D simulations yielded almost identical results. However, at higher Reynolds numbers (Re = 1000), strong three-dimensional effects emerged. Three response branches of wake vortices were identified, with the upper branch exhibiting the strongest 3D characteristics and the initial branch the weakest. Changes in vortex shedding modes directly influenced the lift coefficient, emphasizing the importance of 3D effects.

Martini et al. [51] also performed numerical simulations of cylinder VIVs and compared the results of 2D and 3D URANS models. Consistent with Zhao et al.’s [50] experimental findings, 2D simulations exhibited limitations at high Reynolds numbers, particularly in the prediction of dimensionless vibration amplitudes in the upper branch. The 3D simulations, by contrast, more accurately matched experimental data and effectively addressed the shortcomings of 2D models.

When pursuing prey, harbor seals can reach speeds of up to 2 m/s. At such velocities, the whiskers of other animals would typically experience significant oscillations due to the influence of the Kármán vortex street, resulting in pronounced vortex-induced vibrations (VIVs) through interactions with the surrounding flow. For pinnipeds such as seals that rely on their whiskers for underwater hydrodynamic sensing, self-induced VIVs could interfere with the tracking of flow signals, thereby introducing unnecessary background noise. However, empirical studies have shown that at a swimming speed of 1 m/s, seal whiskers exhibit lateral vibrations of less than 2 mm—barely perceptible to the naked eye [17]. This finding indicates that the undulated morphology of seal whiskers offers significant advantages over simple cylindrical or elliptical geometries.

In the field of underwater sensing, vortex-induced vibrations are a common phenomenon. On one hand, VIVs have several adverse effects: the periodic shedding of vortices applies lateral forces on the sensor structure, and if the structure’s natural frequency coincides with the frequency of these periodic loads, it can lead to strong resonant vibrations. Such vibrations may disrupt the normal functioning of the sensor and reduce measurement accuracy. Additionally, VIVs introduce noise into the sensor’s output signals, which can obscure useful information and complicate signal processing. Long-term exposure to VIVs may also cause structural damage such as cracks, thereby reducing the sensor’s operational lifespan.

On the other hand, most underwater targets generate Kármán vortex streets downstream as they move through fluid. Thus, VIVs can be leveraged to detect these flow disturbances, making it feasible to assess the characteristics of vortex shedding and guide sensor optimization.

Therefore, the development of sensors that can suppress self-induced VIVs while remaining highly sensitive to the vortex streets generated by external targets holds significant practical value. To ensure the stability and reliability of such sensors, the design must consider the potential impact of VIVs, and the sensor geometry should be modified to reduce the formation of vortex streets around the structure. This ensures that the sensor can provide accurate and dependable results during underwater detection tasks. Remarkably, biological studies have shown that seal whiskers naturally fulfill these requirements, offering a compelling bioinspired model for advanced sensor design.

## 4. The Excellent Vortex-Induced Vibration Suppression Mechanism of Seal Whiskers

Marine mammals typically possess streamlined body shapes that minimize hydrodynamic drag, while their appendage structures exhibit unique advantages in modulating drag, lift, thrust, and suppressing vortex-induced vibrations (VIVs). Pinnipeds, in particular, have highly flexible flipper geometries that enhance thrust generation and enable efficient propulsion. For example, the leading-edge tubercles of the humpback whale (*Megaptera novaeangliae*) [52] flippers and the dorsal fins of the harbor porpoise (*Phocoena phocoena*) [53] have been demonstrated to separate spanwise vortical structures, reduce fluid resistance, and mitigate interactions between the body and the surrounding flow field—thus effectively suppressing VIVs and improving hydrodynamic performance.

Additionally, the whiskers of pinnipeds such as the harbor seal (*Phoca vitulina*) are widely recognized as one of the most effective natural structures for suppressing VIVs. As detailed in Section 2, harbor seal whiskers exhibit a characteristic sinusoidal geometry with periodic wave crests and troughs, which disrupt the formation of Kármán vortex streets and regulate lift and drag forces [16]. To further explore the impact of different geometrical structures on the strength of VIVs and highlight the advantages of seal whiskers in vibration suppression, numerous studies have employed techniques such as particle image velocimetry (PIV) and computational fluid dynamics (CFD) to systematically investigate the underlying mechanisms.

### 4.1. Comparative Analysis of the Role of Seal Whiskers and Other Structures in Suppressing Vortex-Induced Vibrations (VIVs)

**Figure 7:**
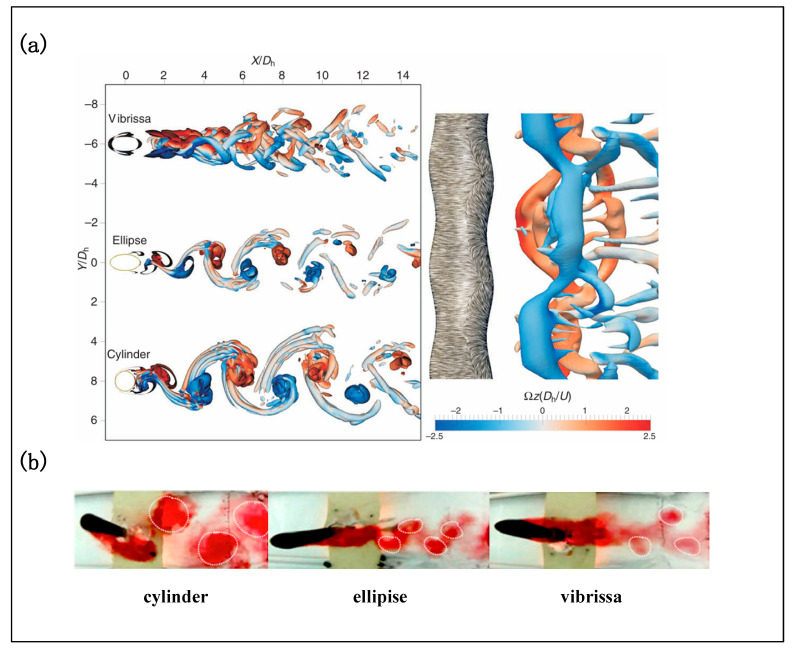
Comparison of the effects of different structures on the suppression of vortex-induced vibration. (**a**) Vorticity in the wake of a cylinder, an elliptical cylinder, and a harbor seal whisker [17]. (**b**) Vortices in the wake of a cylinder, an elliptical cylinder, and a harbor seal whisker [32].

Hanke et al. [17] combined PIV and CFD to examine the dynamic response of seal and sea lion whiskers under similar hydrodynamic tracking conditions. The animals were matched in body size, age, and Reynolds number to isolate whisker morphology as the primary variable. The results revealed significant differences in wake structures: compared with typical cylindrical bodies, harbor seal whiskers exhibited reduced primary vortex shedding, more complex three-dimensional vortices, downstream-shifted shedding zones, and a more symmetric pressure distribution in the wake. Quantitatively, lift was reduced by over 90%, and mean drag was lowered by approximately 40%. The discrepancy between simulated Reynolds stresses and experimental data was within ±30%. Notably, the whiskers of harbor seals were at least six times more effective in suppressing self-induced VIVs compared to those of California sea lions. The observed vibration suppression was attributed to (i) disruption of vortex street formation due to the periodic sinusoidal geometry, (ii) delayed onset of flow instability away from the whisker surface, and (iii) a more symmetric pressure field that promotes uniform force distribution. These results confirm the strong anti-VIV performance of the undulatory seal whisker structure (Figure 7a).

To further elucidate the hydrodynamic advantages of seal whiskers under various flow conditions, researchers have conducted extensive simulation and experimental studies [54,55]. CFD simulations and flow visualization revealed distinct vibration modes and wake evolution mechanisms in response to different flow velocities and upstream disturbances. Physical modeling and scaled-up tank experiments using biomimetic whisker replicas were also conducted to validate theoretical predictions. These findings not only deepen our understanding of the flow-sensing capabilities of seal whiskers but also offer valuable insights for the development of underwater biomimetic sensors [56].

Miersch et al. [54] modified the diameter of an upstream cylinder to simulate fish-like wake flows and studied the downstream whisker responses of seals and sea lions. Both animals could detect the vortex shedding frequency within a flow velocity range of 180–450 mm/s, with a detection error below 30%. However, seal whiskers exhibited significantly higher signal-to-noise ratios (SNRs) and were an order of magnitude more effective in VIV suppression than sea lion whiskers.

Beem et al. [40] employed dye visualization to examine wake structures generated by different geometries. Compared with traditional cylindrical structures, biomimetic whiskers produced no large-scale vortices, and the vortex cores were positioned further downstream (Figure 7b). The vibration frequency synchronized with the vortex shedding frequency of the upstream cylinder and exhibited strong correlation with the Strouhal number (St). In free-stream flow, the whisker oscillation amplitude was minimal, whereas it increased substantially in vortex street-dominated wakes.

Bunjevac et al. [18] used PIV to compare the wake turbulence intensity of walrus whiskers (undulated) and California sea lion whiskers (smooth) at various Reynolds numbers. Both structures reduced vortex shedding frequency, but the undulated geometry outperformed the smooth variant. Specifically, the velocity power spectral density of the undulated whisker wake was approximately 40% lower than that of the smooth whisker, indicating improved flow stability.

While Schulte-Pelkum et al. [24] reported that only ~30% of seals exhibit oscillatory swimming paths during hydrodynamic tracking—compared to ~70% following straight paths—this has led to ongoing debate regarding the essentiality of undulated whiskers. Some researchers argue that California sea lions, which lack pronounced undulations, can still hunt effectively in complex underwater environments. However, under turbid and low-visibility conditions, such as in deep-sea environments, flow perturbations caused by prey become critical sensory cues. Studies have shown that when visual acuity is impaired, seals increasingly rely on vibrissal flow sensing [57]. The sinusoidal geometry of harbor seal whiskers thus functions as a redundant yet highly sensitive mechanosensory system that enhances prey detection and localization.

Witte et al. [55], building upon the seven structural parameters identified by Hanke et al., conducted stereo-PIV and CFD studies to investigate the wake and force characteristics of seal whiskers. They found that the whisker’s sinusoidal spanwise distribution and inclined elliptical cross-sections effectively modulate shear-layer roll-up and reduce lift and drag fluctuations. CFD results (Re = 500) showed that the whisker wake lacked typical Kármán vortex structures and exhibited delayed shedding, with the formation of primary and secondary counter-rotating vortices and highly curved separation lines. The drag coefficient was reduced by ~40%, and force fluctuations were significantly diminished. Proper orthogonal decomposition (POD) further revealed that vortex formation was delayed and decomposed more rapidly in the wake, resulting in a 90% reduction in fluctuating lift compared to cylindrical bodies. Similarly, Chen et al. [58] demonstrated that the biomimetic surface suppressed large-scale vortex formation, promoted small-scale turbulence, and disrupted the periodicity of vortex shedding, thereby enhancing the three-dimensionality of the wake.

Kamat et al. [38], using finite element analysis, found that the VIVs suppression effect strongly depended on the dimensionless ratio of wave crest spacing to mean whisker diameter (λ/D_m_), with the optimal range identified as 4.4–4.6. Song et al. [56,59] employed CFD to compare the VIVs responses of whisker-shaped, cylindrical, and elliptical structures in uniform flow. Results showed that the whisker-shaped configuration exhibited the smallest vibration amplitude and the best overall performance in drag and vibration reduction.

In summary, a growing body of experimental and numerical studies has confirmed the structural superiority of harbor seal whiskers in suppressing VIVs. Their undulatory geometry, vortex disruption capabilities, and pressure field modulation represent a bioinspired flow control strategy with considerable potential for engineering applications. Table 3 systematically summarizes representative studies by Hanke, Beem, Witte, and others, highlighting key findings, methodologies, and performance metrics relevant to VIVs mitigation using seal whisker structures.

### 4.2. Effect of Angle of Attack (AOA) on the VIV Suppression Performance of Seal Whiskers

As demonstrated in the preceding section, the wavy surface structure of harbor seal vibrissae (HSV) exhibits pronounced suppression of vortex-induced vibrations (VIVs) in uniform flow conditions. In addition to the vibrissae geometry, the angle of attack (AOA) between the whisker and the flow direction plays a crucial role in modulating its hydrodynamic response. Studies have shown that as the AOA increases from 0°, the effectiveness of VIVs suppression gradually decreases [60]. This phenomenon is primarily attributed to the elliptical cross-section of the whiskers: changes in AOA alter the projected frontal area, thereby modifying the flow-induced forces and influencing the whisker’s VIVs behavior. This section discusses the influence of AOA variation on the VIVs suppression performance of seal whiskers.

**Figure 8:**
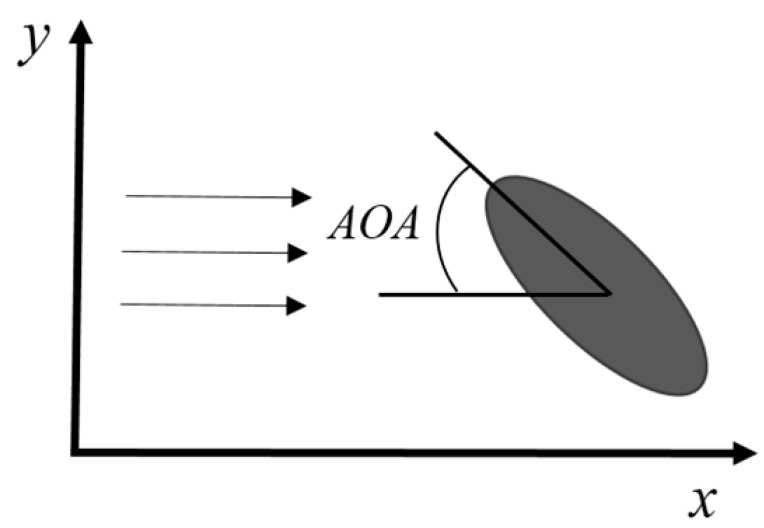
Schematic diagram of angle of AOA.

In this context, AOA refers specifically to the angle between the long axis of the whisker’s elliptical cross-section and the direction of incoming flow, as illustrated in Figure 8. AOA strongly affects lift, drag, and wake stability. Behavioral observations further indicate that seals actively control their vibrissae orientation to maintain a near-zero AOA during prey tracking, thereby optimizing sensory accuracy [17]. This highlights the sensitivity of vibrissal wake dynamics to changes in AOA.

Previous studies have revealed that both the vibrational amplitude and frequency of whiskers in flow vary with their orientation. Notably, when the broad side of the whisker faces the flow, the vibration frequency tends to increase. Theoretical analyses and behavioral experiments suggest that the wavy geometry enhances hydrodynamic signal detection.

**Figure 9:**
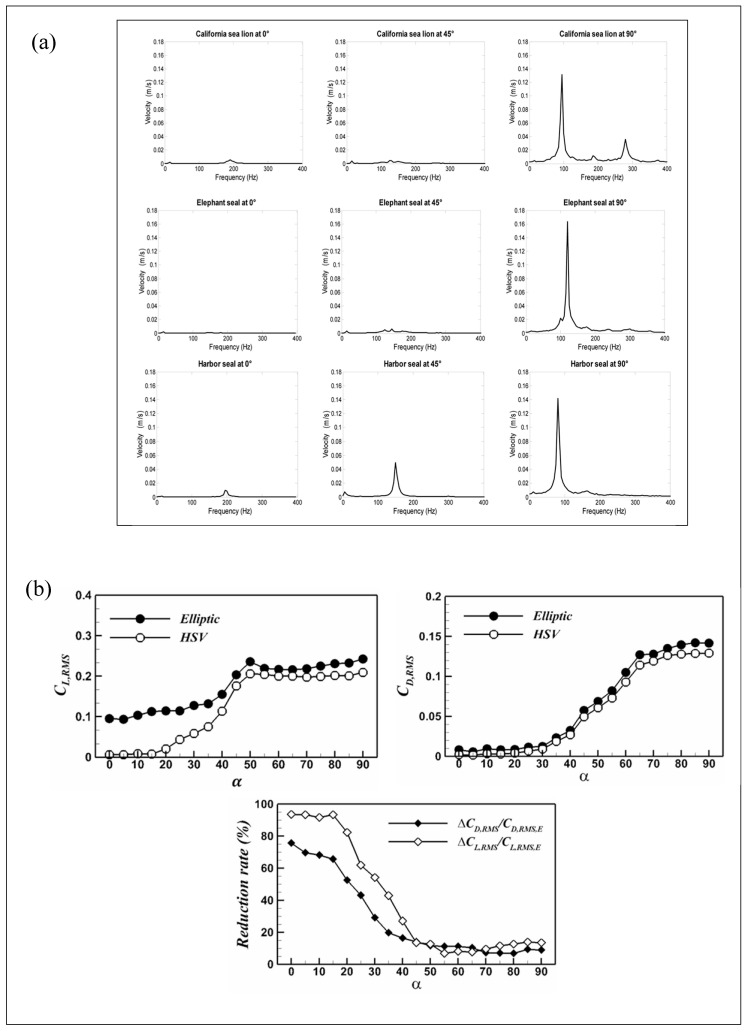
Comparison of the effects of different structures on vortex-induced vibration suppression at different angles of attack. (**a**) Frequency–velocity distribution of California sea lions, elephant seals, and harbor seals [36]. (**b**) Relationship between the root mean square value (RMS) of the force coefficient of the elliptical cylinder and seal whisker structure at different angles of attack and the force reduction ratio of the HSV to the elliptical cylinder [60].

Murphy et al. [36] conducted comparative experiments on wavy (harbor seal and walrus) and smooth (California sea lion) whisker structures under varying AOAs to investigate flow-induced vibration (a broader category encompassing VIVs). While the structural differences had negligible effects on vibration frequency and velocity, AOA was found to significantly influence both. Specifically, at 0° AOA, vibration velocity was minimized and frequency maximized, while at 90°, the opposite trend was observed (Figure 9a). These findings suggest that seals may actively minimize AOA to reduce self-induced noise during swimming. A similar conclusion was drawn by Song et al. [56], who reported that whisker vibration amplitude increased with rising AOA under constant flow conditions.

Bunjevac et al. [18] provided further evidence that AOA strongly influences wake topology and turbulence. At 0° AOA, the reverse flow region was small, vortex decay was rapid, and turbulence intensity was significantly reduced, all of which contributed to enhanced VIV suppression. However, at 90° AOA, the wake region expanded, the flow velocity deficit increased, and vortex persistence was observed even 25D_h_ downstream. These results underscore that seal whiskers are most effective in VIV mitigation at low AOA, with performance deteriorating significantly at higher angles.

Prior work by Witte [55] and Wang [61] had already demonstrated that the wavy surface geometry of whiskers promotes favorable vortex separation. This is achieved by generating symmetrically shed counter-rotating vortices, which in turn reduce drag and suppress vibration amplitudes.

Wang and Liu [61] employed PIV techniques to study the wake dynamics of seal whiskers at Re = 1800 under various AOAs and compared the results with those from cylinders and elliptical rods of identical hydraulic diameter. The results revealed that the seal whisker model generated smaller and more stable recirculation zones, with significantly reduced streamwise and spanwise velocity fluctuations. Moreover, seals were observed to control AOA within a functional range of approximately −30° to +30° during natural locomotion.

In a subsequent study, Wang et al. [62] used time-resolved PIV (TR-PIV) to investigate VIV characteristics of whiskers at AOAs of 0°, 30°, 60°, and 90° at Re = 1800 in a wind tunnel setting. They found that vibrational responses were negligible for AOAs ≤ 30° but increased substantially at higher AOAs.

Despite the importance of AOA in regulating whisker-induced VIVs, comprehensive studies remain limited. Most experimental investigations—including those by Hanke [17], White, Murphy [36], Bunjevac [18], and Wang [62]—have been restricted to discrete angles (e.g., 0°, 30°, 45°, 60°, 90°). To address this gap, Kim and Yoon [60] expanded the tested AOA range to 0–90° and compared HSV and elliptical cylinders in terms of shedding frequency and force coefficients. Their results showed that the vortex shedding frequency for elliptical cylinders decreased monotonically with increasing AOA, whereas HSV displayed a non-monotonic trend—initially increasing and then decreasing. Regarding force coefficients, the lift coefficient (C_L_) peaked at AOA ≈ 50°, and the drag coefficient (C_D_) reached a maximum at 90° (Figure 9b), with HSV consistently exhibiting lower values than elliptical cylinders.

Zhao et al. [63] investigated vibrational responses, frequencies, and fluid forces on whiskers at AOAs ranging from 0° to 90°. In the wake of a cylinder, whiskers exhibited a combination of VIV and wake-induced vibration (WIV). In the presence of caudal fin wake flow, they exhibited VIV–WIV coupling for AOA < 45°, and VIV–wake-induced flapping for AOA > 60°. These findings indicate that whisker response modes are highly AOA-dependent.

Assi and Bearman [64] experimentally compared wavy cylinders and smooth cylinders with identical diameters over a wide Reynolds number range (1500–15000). They analyzed fluid force decomposition and conducted wake visualization to assess vibration mechanisms. Interestingly, at α = 0° and α = 45°, both cylinder types exhibited similar vibration amplitudes and frequencies, suggesting that not all geometric modifications are effective for VIV suppression. This result reinforces the unique functional significance of seal whisker geometry in flow control applications.

In conclusion, the angle of attack plays a pivotal role in modulating the hydrodynamic performance of seal whiskers. Table 4 is a comparison of the inhibitory effect of seal whiskers on vortex-induced vibration at different attack angles made by researchers. While the wavy geometry inherently supports VIVs suppression, its efficacy is highly sensitive to AOA. Future studies should consider a broader and more continuous AOA range, coupled with high-resolution flow diagnostics, to better understand the dynamic coupling between structure, orientation, and flow-induced forces.

In the preceding sections, various researchers have investigated the structural advantages of harbor seal whiskers in VIVs from different perspectives. These studies employed a range of approaches, including particle image velocimetry (PIV), computational fluid dynamics (CFD) simulations, finite element analysis (FEA), and comparative experiments using biomimetic structures. Each method offers unique advantages and faces distinct limitations, as summarized in Table 5.

Furthermore, there remains ongoing debate among researchers regarding the key mechanisms by which whisker morphology contributes to vibration suppression. Hanke et al. [17] and Witte et al. [55] emphasized that the sinusoidal geometry of crests and troughs is central to inhibiting vortex shedding and stabilizing lift–drag fluctuations. However, it is worth noting that California sea lions, despite lacking such wavy whisker structures, still demonstrate strong VIV suppression capabilities. This suggests that additional or alternative mechanisms may also be involved.

A comprehensive understanding of the vibrational responses and fluid force characteristics of seal whiskers across varying wake environments and angles of attack not only enhances our knowledge of VIVs suppression mechanisms, but also provides valuable theoretical and empirical support for engineering applications. Building upon these findings, recent research efforts have begun to translate bioinspired whisker geometries into practical vibration suppression strategies for real-world engineering systems.

### 4.3. Engineering Applications of Seal Whisker-Inspired Structures in VIVs Suppression

**Figure 10:**
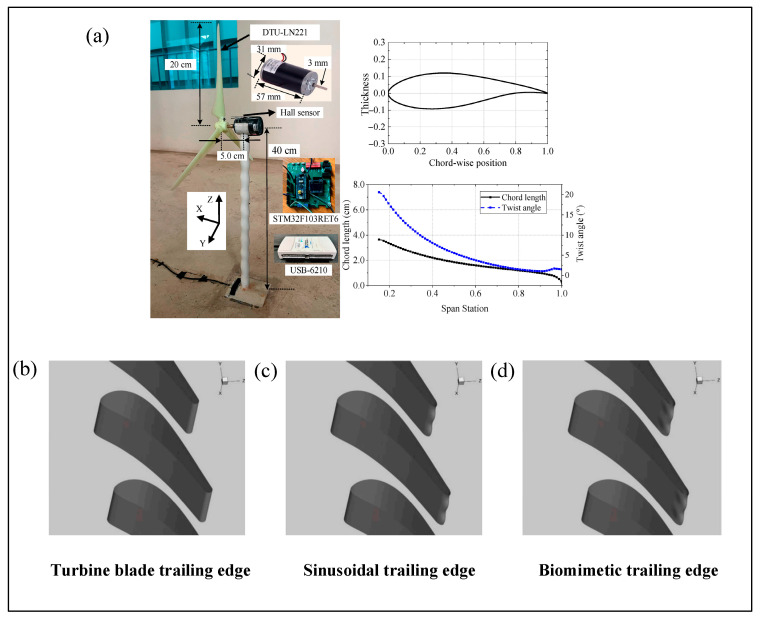
Overview. (**a**) Bionic whisker structure wind turbine pile [71]. (**b**) 3D view of the trailing edge of a conventional turbine blade [72]. (**c**) 3D view of the trailing edge of a sinusoidal turbine blade [72]. (**d**) 3D view of the trailing edge of a bionic whisker-shaped turbine blade [72].

In practical engineering, the suppression of vortex-induced vibrations (VIVs) remains a major challenge in structural dynamics and fluid–structure interaction research. Over the past few decades, extensive investigations have been conducted to mitigate VIVs in various industrial scenarios [65,66,67]. For example, in common structures such as bridge piers and offshore wind power foundations, VIVs may cause fatigue damage, decreased stability, and even structural failure. The introduction of seal whisker-like structures provides a new solution for vibration reduction design. Figure 10a shows a micro-bionic whisker structure wind power pile.

Studies have demonstrated that wavy-surfaced geometries modeled after seal whiskers can significantly disrupt vortex street formation, thereby reducing periodic loading and enhancing structural stability and fatigue life, while minimizing maintenance costs. For instance, in offshore oil drilling platforms, engineers have adopted undulated base structures inspired by whisker geometry to suppress VIVs. Similarly, in turbomachinery design, biomimetic trailing-edge structures resembling seal whiskers have been employed to optimize aerodynamic performance. These structures have shown notable advantages in wake flow control, suppression of rotor–stator interference, and reduction of aerodynamic losses. By mitigating VIV-induced noise and fatigue, such designs contribute to higher operational efficiency and extended service life of turbine blades. It has been reported that whisker-inspired trailing edges can reduce aerodynamic drag by up to 50% [68]. Figure 10b–d illustrates the 3D morphology of conventional, sinusoidal, and whisker-mimicking trailing edge geometries.

In real-world applications, traditional VIV suppression techniques—such as helical strakes—are effective but often result in increased drag. In contrast, biomimetic surface modifications offer a more efficient alternative. For instance, applying sinusoidal patterns to the leading edge of rectangular-section bodies has been shown to reduce drag by approximately 30% [69], while implementing undulated patterns in the inflow direction of cylindrical bodies can reduce the mean drag coefficient by about 20%, effectively mitigating the formation of classical Kármán vortex streets [60,70].

Moreover, in underwater sensing applications requiring high precision, conventional smooth cylindrical sensors tend to generate self-induced noise due to VIVs, compromising detection accuracy. By contrast, sensors incorporating seal whisker-inspired geometries benefit from their periodic undulated surfaces, which suppress self-excited vibrations and enable more accurate detection of subtle flow disturbances—such as wake vortices from nearby objects—during underwater navigation. This improves the sensitivity, resolution, and reliability of flow field perception, offering promising potential for advanced underwater target tracking and autonomous guidance systems.

## 5. Advances in Bioinspired Seal Whisker Sensors

**Figure 11:**
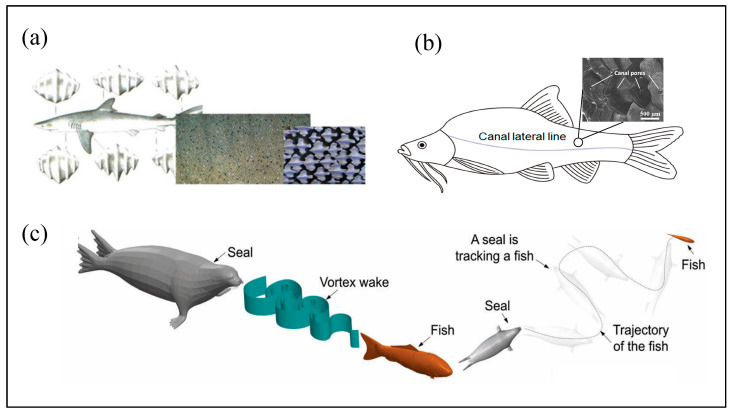
Overview. (**a**) Microscopic structure of shark skin [73]. (**b**) Schematic diagram of the lateral line system of fish [82]. (**c**) Schematic diagram of the track created by a seal chasing a fish [83].

The unique morphological structure of marine organisms has provided rich design inspiration for the development of bionic technology and promoted research in the intersection of biology and marine engineering. For example, the surface of shark skin has a rich micro-texture structure [73] (Figure 11a), which can effectively reduce attached biological pollution and fluid resistance. If its bionic structure is applied to the surface of the hull, it can significantly reduce navigation resistance and improve propulsion efficiency [74,75]. The lateral line system of fish shown in Figure 11b is a mechanical sensory organ that can sense changes in the surrounding water flow and assist in foraging, obstacle avoidance, migration, and reproduction [76,77,78]. The microelectromechanical system (MEMS) artificial lateral line developed based on this principle can achieve high-precision detection of flow speed and direction [79,80,81]. Studies have found that even if the sight of the spotted seal is blocked, it can rely on its whiskers to accurately identify the wake disturbance caused by prey swimming in the water (Figure 11c) [21,22,23,24].

Extensive studies have demonstrated that the undulated surface geometry of seal whiskers plays a crucial role in suppressing vortex-induced vibrations (VIVs) [17,18,40,54]. Motivated by these findings, researchers have proposed and developed various biomimetic seal whisker-inspired sensors for flow detection and vortex tracking applications. These bioinspired flow sensors typically mimic the structural principles of the follicle–sinus complex (FSC) found in natural vibrissae, comprising a high-aspect-ratio whisker structure mounted on a flexible sensing base. When exposed to fluid disturbances, the whisker undergoes bending deformation, which in turn induces strain on the base, facilitating the conversion of hydrodynamic forces into measurable mechanical signals.

Depending on the sensing mechanism, current bioinspired whisker flow sensors can be categorized into several types, including resistive, capacitive, piezoelectric, and optical sensors. Each modality offers distinct advantages in sensitivity, bandwidth, and robustness across various fluid sensing scenarios. These innovations have laid a solid foundation for integrating high-resolution, miniaturized flow sensing capabilities into underwater unmanned vehicles (UUVs) and other autonomous marine systems.

### 5.1. Selection and Performance Comparison of Materials for Bionic Seal Whiskers

To enhance the dynamic bionic responsiveness and durability of artificial seal whisker sensors, careful selection of materials for the whisker shaft is essential. Currently, materials such as polydimethylsiloxane (PDMS), photopolymer resins, and silicone-based elastomers are commonly used in bionic whisker fabrication. However, these artificial materials exhibit significant differences in Young’s modulus, fatigue life, and dynamic behavior when compared to natural seal whiskers composed of keratin, as summarized in Table 6.

Designing bionic whiskers requires balancing mechanical performance, fatigue resistance, manufacturing reproducibility, and dynamic compatibility with biological systems. Natural seal whiskers, composed of keratin, exhibit a Young’s modulus of approximately 2–4 GPa and possess fatigue lifespans that often match the full life expectancy of marine mammals (typically 25–35 years). Despite inter-individual biological variability, the undulated morphology of seal whiskers remains a widely accepted template for bionic design due to its role in minimizing fluid disturbance and enhancing sensitivity.

Among synthetic alternatives, polyurethane and photopolymer resins are commonly used in rapid prototyping due to their processability. Polyurethane exhibits a tunable Young’s modulus ranging from 1 to 100 MPa, with favorable flexibility and fatigue resistance, making it suitable for applications involving frequent vibration. Moreover, its high reproducibility in mold-based fabrication supports scalable manufacturing. In contrast, photopolymer resins, despite possessing higher modulus values (1–3 GPa), typically suffer from low fatigue resistance and brittleness, limiting their durability under dynamic loading conditions.

PDMS, widely used in micro/nanofabrication and soft sensor applications, offers a much lower modulus (0.5–3 MPa) but excels in flexibility, chemical stability, and underwater longevity. Studies have shown that PDMS structures can remain mechanically stable for multiple years in aqueous environments, making it particularly well suited for short-cycle use or modular replacement systems.

In summary, flexible polymers such as polyurethane and PDMS present a compelling balance of fatigue resistance and bionic dynamic compatibility. These materials are particularly advantageous in large-scale array configurations of bionic whisker sensors, where structural stability, manufacturing reproducibility, and cost-effectiveness are critical. Future material development should also prioritize functional compatibility with signal transduction systems—including triboelectric, piezoelectric, or fiber-optic readout modules—to achieve optimal system-level performance.

### 5.2. Capacitive Bioinspired Seal Whisker Sensors

Capacitive bioinspired seal whisker sensors utilize capacitors as the primary sensing elements. When the biomimetic whisker experiences mechanical deformation due to external fluid flow, the relative spacing between the capacitor’s parallel plates changes, resulting in a variation in capacitance. By detecting these changes, the sensor can infer the degree of whisker deflection. This type of sensor offers notable advantages, including high sensitivity, low power consumption, and minimal sensitivity to temperature fluctuations [84].

**Figure 12:**
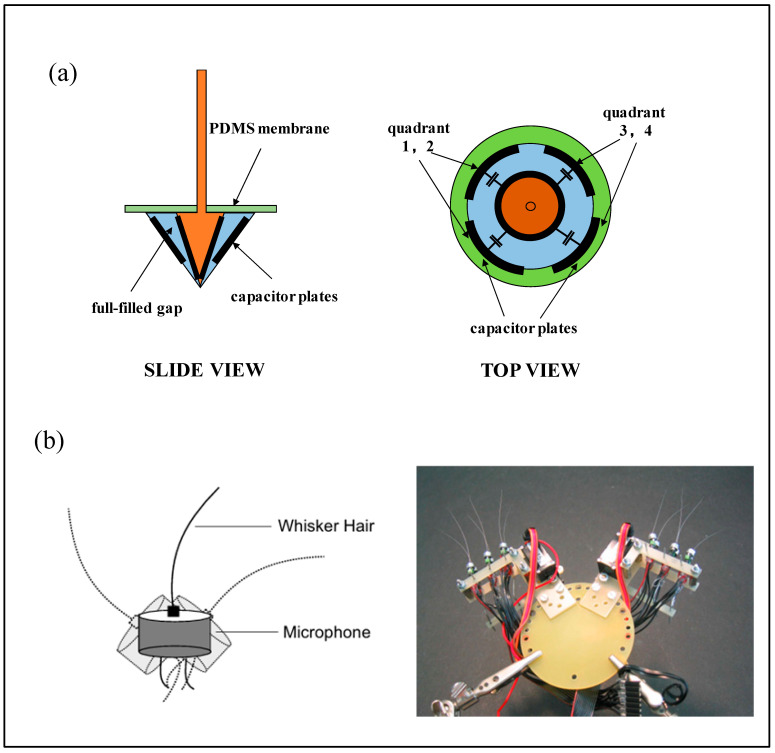
Capacitive bionic seal whisker sensor. (**a**) Capacitive sensor structure. (**b**) Schematic diagram of directly integrated capacitive bionic whisker motion (left) and two whisker capacitor arrays (right) [84].

For instance, one design adopts a conical–sleeve conical parallel-plate capacitor configuration, covered with a thin layer of polydimethylsiloxane (PDMS), which enables detection of fluid motion from four directions within the wake of an object (Figure 12a) [85,86].

Another capacitive sensor design, shown in Figure 12b, integrates the whisker directly into the capacitor base structure [87]. While this approach enables more accurate tracking of high-frequency whisker motion, it does not capture directional information. Owing to its compact form factor, this type of sensor is well suited for array integration and can be effectively deployed in robotic systems for environmental perception in complex flow fields.

It is worth noting, however, that capacitive whisker sensors are generally susceptible to parasitic capacitance and electromagnetic interference, which may negatively affect sensing accuracy and stability.

### 5.3. Resistive Bioinspired Seal Whisker Sensors

Resistive bioinspired seal whisker sensors operate on the principle of resistance variation in response to mechanical deformation. When the whisker is deflected by external flow stimuli, the resulting change in resistance reflects the degree of structural deformation. These sensors feature simple architectures, low power consumption, and broad sensing ranges, and represent a typical fusion of resistive transduction and biomimetic design principles [88].

**Figure 13:**
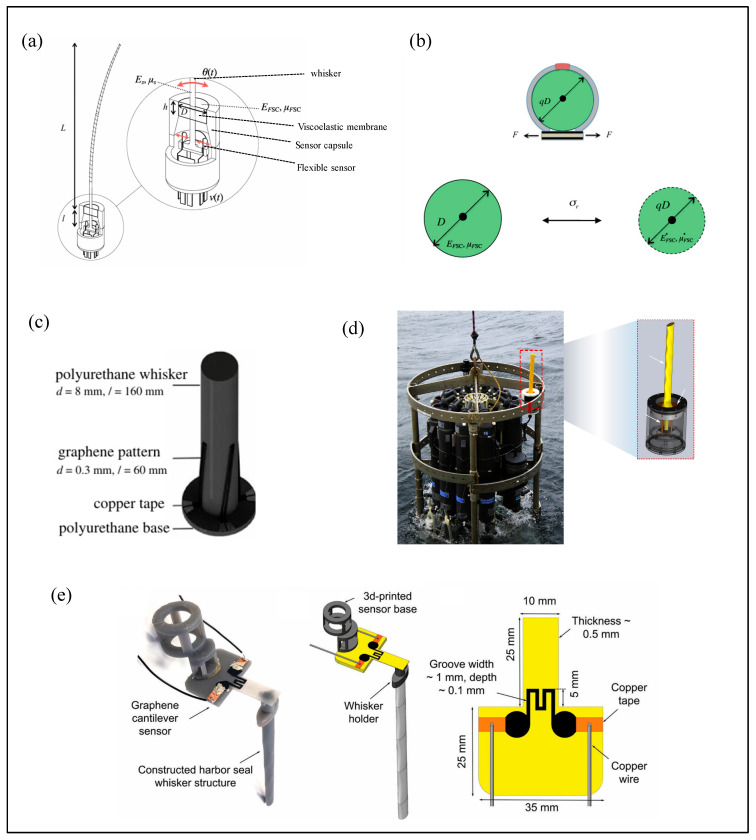
Resistive bionic seal whisker sensor. (**a**) Schematic diagram of bionic whisker sensor [89,90,91]. (**b**) Compression sensing mechanism of hair follicle membrane [89,90,91]. (**c**) 3D-printed flexible sensor proposed by Gul et al. [92]. (**d**) Seal whisker flow sensor developed by Beem et al. [40,93]. (**e**) 3D-printed seal whisker flow sensor [98].

A research team from the Singapore–MIT Alliance for Research and Technology (SMART) [89,90,91] developed a flexible resistive underwater sensor inspired by the follicle–sinus complex (FSC) found in seal vibrissae. The whisker shaft is supported by an FSC unit composed of a rigid follicular shell and an elastic membrane (Figure 13a). The membrane provides torsional stiffness and damping, while the rigid shell anchors the assembly. Four flexible displacement sensors are embedded within the FSC to monitor angular vibrations of the whisker and output voltage signals (Figure 13b).

Gul et al. [92] proposed a 3D-printed flexible resistive sensor for underwater vortex detection. This sensor consists of a polyurethane (PU) whisker and four orthogonal graphene conductive patterns (Figure 13c). When the whisker deforms under flow, the resistance of the graphene patterns changes, resulting in a corresponding voltage signal.

Beem et al. [40,93] developed a resistive seal whisker flow sensor for field testing. Their design features a 3D-printed whisker mounted on a flexible base, with four underlying flex sensors (Figure 13d). Fluid-induced whisker motion causes sensor deflection, leading to resistance changes that are translated into voltage signals, enabling flow velocity estimation.

To further enhance sensitivity and lower detection thresholds, recent studies have explored the use of nanomaterials. Liu et al. (Beihang University) [94] designed a novel resistive whisker sensor composed of a wavy whisker structure integrated with a planar pressure-sensitive resistor. The resistor layer is formed by directing conductive nanocomposite ink into four Ω-shaped microchannels using directional liquid spreading (DLS) technology [95], which allows for rapid, high-precision fabrication of complex non-planar geometries. The sensor demonstrates a flow detection threshold as low as 8 mm/s. Abolpour Moshizi et al. [96] developed a flexible resistive sensor based on vertically aligned graphene nanosheets (VGN), achieving a high sensitivity of 103.91 mV·(mm/s)^−1^ and a minimum flow detection threshold of 1.127 mm/s. It exhibited excellent performance across the 0.1–25 Hz frequency range in underwater applications.

Additionally, a team from the University of Groningen [97,98] proposed a concept design for a 3D-printed seal whisker flow sensor, consisting of an optimized central whisker structure and VGN-based sensing elements located at a cantilever hinge (Figure 13e). The entire sensor is fabricated from photosensitive resin using 3D printing, offering a promising low-cost solution for arrayed underwater flow sensing.

### 5.4. Piezoelectric Bioinspired Seal Whisker Sensors

Piezoelectric sensors operate based on the piezoelectric effect, wherein specific materials generate an electric charge when subjected to mechanical stress. This phenomenon has been widely utilized in force–electrical coupling applications and forms the basis for piezoelectric bioinspired sensor designs [88].

**Figure 14:**
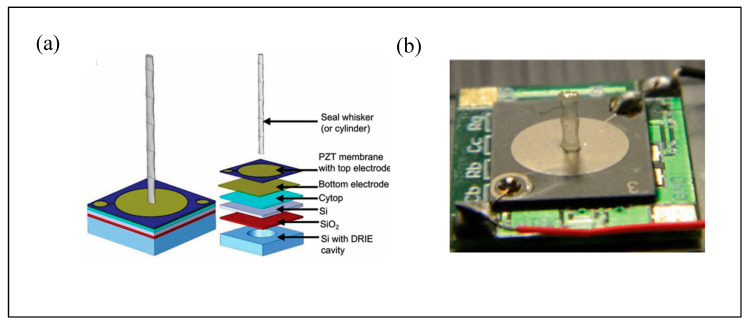
Piezoelectric bionic seal whisker sensor. (**a**) Schematic diagram of piezoelectric bionic seal whisker sensor [38]. (**b**) Actual picture of piezoelectric bionic seal whisker sensor [32].

Kottapalli et al. [38,99,100] developed a piezoelectric MEMS-based seal whisker sensor. The whisker was fabricated using 3D printing, followed by photopolymerization for structural reinforcement, and then mounted onto a PZT (lead zirconate titanate) thin-film substrate. Flow-induced whisker vibrations deform the piezoelectric membrane, generating a measurable voltage output (Figure 14). Figure 14a illustrates the sensor design, while Figure 14b shows the physical prototype. Experimental results indicate a flow detection threshold of 193 μm/s, which is comparable to the hydrodynamic sensitivity of real harbor seal whiskers. In terms of VIV suppression, the sensor demonstrated a performance approximately 50 times greater than that of a cylindrical structure of equivalent dimensions.

Zhang et al. from Harbin Institute of Technology [101] proposed a novel self-powered piezoelectric sensor. The sensor is capable of efficiently detecting both flow angle of attack (AOA) and velocity. Repeated experiments confirmed that the device can differentiate between various AOA conditions and exhibits high velocity sensing sensitivity, with a detection precision reaching 1.445 V·(m/s)^−1^.

### 5.5. Triboelectric Bioinspired Seal Whisker Sensors

In addition to capacitive, resistive, and piezoelectric sensors, researchers have recently integrated triboelectric nanogenerator (TENG) technology—based on triboelectrification and electrostatic induction—into biomimetic seal whisker structures. This integration has led to the development of highly sensitive and flexible triboelectric whisker sensors (TWS) [102]. TENG sensors convert mechanical deformation into electrical signals through contact electrification and subsequent electrostatic induction during material separation, enabling the detection of pressure, motion, vibration, and fluid flow. Compared to piezoelectric sensors, TWSs offer several advantages, including self-powered operation, higher voltage output, and lower manufacturing cost [84].

**Figure 15:**
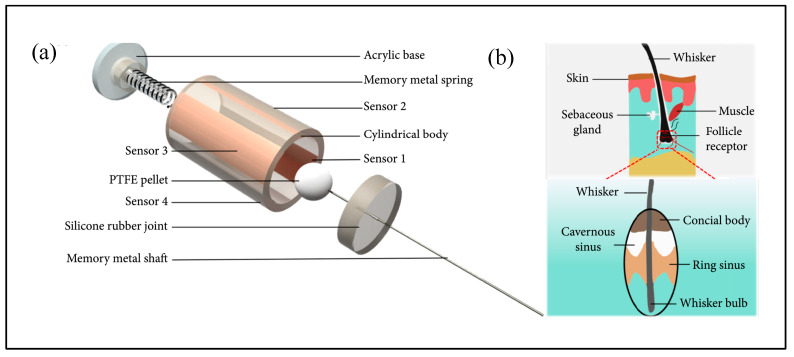
Triboelectric bionic seal whisker sensor. (**a**) Schematic diagram of the structure of the bionic whisker sensor [102]. (**b**) Schematic diagram of the hair follicle structure [102].

A representative TWS design is shown in Figure 15. Figure 15a illustrates the overall structure, while Figure 15b provides a schematic of the follicle-inspired module. The design is inspired by the biological follicle–sinus complex (FSC) and consists of a soft silicone rubber joint, a 3D-printed cylindrical shell, and a base structure. The base contains a spring-loaded polytetrafluoroethylene (PTFE) sphere serving as the dielectric layer, with the spring providing damping and restoring force.

A research team from Dalian Maritime University [103,104] developed an underwater triboelectric whisker sensor by combining TENG technology with seal whisker geometry. The sensor features a TENG-based sensing unit composed of fluorinated ethylene propylene (FEP) film, polyethylene terephthalate (PET) film, and conductive ink. During flow or vortex-induced whisker motion, periodic contact and separation between the FEP film and the conductive layer induce charge transfer, producing measurable electrical signals. Variations in these signals reflect changes in the surrounding hydrodynamic environment, enabling real-time detection of flow disturbances and underwater target tracking.

### 5.6. Optical Bioinspired Seal Whisker Sensors

Although the previously discussed electrical whisker sensors have demonstrated the capability to detect low flow velocity thresholds (as low as 1.127 mm/s), their practical deployment is often limited by challenges such as waterproofing, high system cost, and vulnerability to electromagnetic interference. Optical sensors, by contrast, offer excellent electromagnetic immunity, high sensitivity, miniaturization potential, and superior environmental durability, making them increasingly attractive for bioinspired flow sensing applications.

Wang et al. from Harbin Engineering University [105] designed an underwater whisker sensor based on orthogonally distributed fiber Bragg gratings (FBGs). The FBGs were symmetrically arranged around the artificial whisker structure, allowing the sensor to detect hydrodynamic disturbances within a range of up to 300 mm. The measured wavelength shift sensitivity was reported as 0.86 nm·(m/s)^−1^, demonstrating robust and stable response performance.

**Figure 16:**
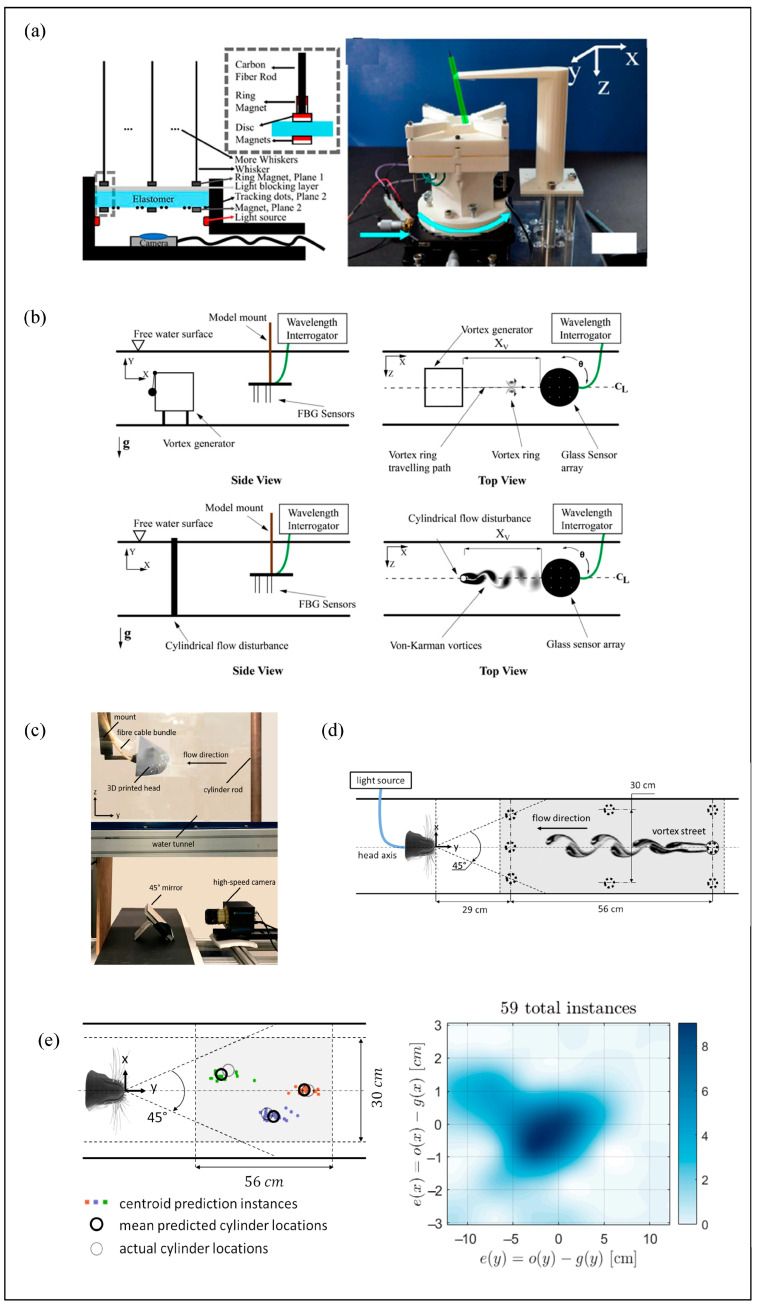
Overview. (**a**) Schematic diagram of the sensor and the experimental setup [86]. (**b**) Fiber Bragg grating (FBG) sensor array [106]. (**c**) Bionic seal head experimental setup [107]. (**d**) Schematic diagram of the water tunnel experimental setup [107]. (**e**) NNS prediction results [107].

Recognizing the advantages of whisker arrays over single sensors—in terms of sensitivity, spatial resolution, and redundancy—researchers proposed an optical array-based sensing method. As illustrated in Figure 16a, a whisker array system was developed by embedding magnets at the base of each whisker, which were mounted on a stretchable membrane with tracking markers. High-speed cameras were employed to capture whisker deflection trajectories in real time, and image processing algorithms extracted multidimensional data for precise flow field detection. The figure also shows the experimental setup and the array design layout.

Glick et al. [106] introduced an improved method for enhancing the sensitivity of FBG-based whisker arrays via fiber-optic strain sensing. The enhanced system demonstrated significantly higher responsiveness to bending stress compared to conventional electrical sensors, as shown in Figure 16b, confirming the potential of optical technologies in building miniaturized and high-performance underwater sensing platforms.

More recently, the integration of bioinspired sensing with artificial intelligence has emerged as a promising direction. Elshalakani et al. [107] developed a deep learning-enhanced whisker sensor composed of spatially distributed optical fibers that emulate the structural pattern of seal vibrissae (Figure 16c–e). Through supervised learning, the system associated whisker vibration data with upstream cylinder positions, enabling accurate localization of wake generators. This approach opens new avenues for intelligent underwater detection and autonomous flow sensing.

A comprehensive review of biomimetic seal whisker sensor studies identifies several geometric parameters as critical to sensing performance: (i) Diameter—natural seal whiskers have diameters on the order of tens of micrometers, whereas biomimetic whisker sensors typically use diameters around 1–1.5 mm to amplify root displacement and enhance sensitivity [108]; (ii) Length and aspect ratio—natural whiskers range from 8 cm to 10.5 cm [32], and sensor designs often employ high-aspect-ratio structures (length/diameter ≈ 6–10) to improve response magnitude and frequency resolution [109]; (iii) Corrugated profile and wavelength—seal whiskers exhibit elliptical cross-sections with periodic corrugations, and their wavelength-to-diameter ratio (λ/D ≈ 4–5) is believed to be evolutionarily optimized—studies show an optimal λ/D ≈ 4.4–4.6 that can reduce vortex-induced vibrations by an order of magnitude and significantly improve signal-to-noise ratio [38]; (iv) Surface micro-texture—though still preliminary in capacitive biomimetic sensors, existing research indicates that micro-textures can stabilize boundary layers and further reduce turbulence-induced noise. Overall, diameter and aspect ratio have the greatest impact on sensitivity; corrugated structure is key to vibration suppression; and surface texturing represents the future direction for improving detection precision. It is recommended that future designs incorporate parameter sweeps and geometric fitting—particularly, tuning λ/D, diameter, and aspect ratio—to maximize biomimetic sensor performance.

Furthermore, to investigate the impact of varying Reynolds numbers on sensor performance, multiple experimental and numerical studies have assessed whisker-inspired sensors across laminar and turbulent regimes. Kim et al. [110] evaluated a single seal whisker in a low-turbulence flume (flow speed 0.17–0.52 m/s, corresponding to Re ≈ 500–2200), observing stable vibration frequencies (48–193 Hz) with a high signal-to-noise ratio (SNR > 7 dB). In vortex-shedding environments established in towing tanks and water channels, the biomimetic whisker consistently locked onto the vortex frequency within the 100–300 Hz range. Furthermore, in channel flow array experiments at U ≈ 0.3 m/s (Re > 5000), the whisker array demonstrated coherent response behavior [108]. Whether utilized as single elements or as part of an array, the whiskers maintained high sensitivity and SNR under both laminar and turbulent conditions. Under laminar flow, the vibration amplitude increased linearly with velocity, while in turbulent vortex-shedding scenarios, the vortex frequency was reliably detected.

In today’s context, the scientific and efficient exploration of complex marine resources remains a global challenge. Conventional underwater sensing techniques, such as acoustic detection and optical imaging, face inherent limitations. Acoustic systems may disrupt the natural behavior of aquatic organisms, while optical methods are highly susceptible to environmental factors such as turbidity, leading to significant signal attenuation. In this regard, bioinspired seal whisker sensors—renowned for their high sensitivity and signal-to-noise ratio—have attracted increasing attention as core sensing components for next-generation high-precision underwater detection systems. Compared to standalone acoustic or optical approaches, biomimetic flow-sensing technologies offer enhanced robustness and adaptability to complex environments, holding great promise for future marine exploration applications.

## 6. Conclusions and Future Prospects

This review systematically summarized the critical role of seal whiskers—particularly, those of the harbor seal—in underwater hydrodynamic sensing. Studies have demonstrated that seals can identify vortex wake signals generated by moving prey to estimate their size and trajectory. Based on this biological capability, various research teams have quantified whisker structures using 2D imaging and 3D scanning reconstruction techniques.

Building on the structural modeling, the review introduced the fluid–structure interaction (FSI) framework, including discussions on the formation of Kármán vortex streets and the mechanisms of VIVs. Seal whiskers exhibit superior performance in suppressing VIVs compared to traditional cylindrical structures, which has inspired the development of multiple biomimetic whisker sensors. This review highlighted five major categories of sensors—capacitive, piezoelectric, resistive, triboelectric, and optical—focusing on their structural designs and functional advantages.

With the rise of intelligent marine platforms, such as underwater robots and unmanned underwater vehicles (UUVs), there is an increasing demand for high-sensitivity, high-accuracy flow sensing technologies. The distinctive undulated morphology of seal whiskers offers excellent noise suppression and a high signal-to-noise ratio in complex aquatic environments, making biomimetic whisker sensors particularly well suited for precise flow field sensing.

As next-generation sensory components, bioinspired seal whisker sensors provide robust navigation, obstacle avoidance, and target-tracking capabilities for underwater robotic systems. Looking ahead, these sensors could also enhance signal stability and anti-interference performance in underwater acoustic devices. Their advantages—high sensitivity, low power consumption, and scalability—open new avenues for marine environmental monitoring, resource exploration, and biomimetic robotics, offering significant promise for the future of ocean technology.

## Figures and Tables

**Table 1:** Representative experiments on hydrodynamic trail tracking by harbor seals.

Researchers	Methodology	Key Findings	Key Data/Parameters	Ref.
Renouf et al.	Trout prey capture behavioral experiment	Seals without whiskers failed to effectively capture prey; first proposed sensory function of whiskers	N/A	[21]
Dehnhardt et al.	Vibrating sphere stimulation + sensory blocking	Seals can detect weak hydrodynamic disturbances using whiskers	Sensitivity threshold > 50 Hz; response range: 10–100 Hz	[4]
Dehnhardt et al.	Mini-submarine trail tracking experiment	Seals tracked hydrodynamic trails produced up to 30 s earlier; demonstrated vortex wake tracking ability	Detectable flow speed: 16 mm/s	[22]
Schulte-Pelkum et al.	Real seal tracking + PIV visualization	Identified two tracking modes: linear and oscillatory	64% linear tracking; 34% oscillatory tracking	[24]
Wieskotten et al.	Drag-induced wake recognition + PIV	First confirmed that seals can judge object direction, size, and shape via reverse vortex patterns	Whiskers can resolve spatial structures in wakes	[25,26]
Murphy et al.	Vibration frequency stimulation experiment	Seals are sensitive to frequencies between 20 and 250 Hz; optimal response around 80 Hz	Optimal frequency response: ~80 Hz	[30]

**Table 2:** Comparative analysis of geometric models of harbor seal whiskers proposed by Hanke, Ginter, Murphy, Rinehart, and Kamat.

Harbor Seal	2a	2k	Peak to Peak	Trough to Trough	Ref.
Hanke	1.19	095	1.82	N/A	[17]
Ginter	0.92 ± 0.13	0.73 ± 0.12	3.27 ± 0.39	3.26 ± 0.40	[35]
Murphy	1.11 ± 0.08	0.879 ± 0.03	3.88 ± 0.45	N/A	[36]
Rinehart	1.05 ± 0.24	0.83 ± 0.19	3.44 ± 0.72	3.45 ± 0.73	[37]
Kamat	1.17 ± 0.10	0.93 ± 0.19	3.49 ± 0.33	3.53 ± 0.33	[38]

**Table 3:** Representative studies on the role of seal whiskers in suppressing vortex-induced vibrations (VIVs).

Researchers	Methodology	Key Findings	Metrics/Conclusions	Ref.
Hanke et al.	PIV + CFD (comparison between seal and sea lion)	Whisker structure delays vortex street formation, improves wake symmetry	Lift reduced by 90%; drag reduced by 40%; >6× suppression vs. sea lion	[17]
Miersch et al.	Experiment (control upstream cylinder size)	Whiskers accurately detect wake frequencies; significantly improved SNR	Frequency detection error < 30%; >10× VIV suppression vs. sea lion	[54]
Beem et al.	Dye visualization + amplitude/frequency testing	Whisker wakes show no large vortices; vortices remain far from body surface	Minimal vibration amplitude in free flow; significantly increased in wake	[40]
Bunjevac et al.	PIV (comparison: wavy vs. smooth whiskers)	Wavy whiskers reduce wake turbulence and vortex shedding frequency	Power spectral density ~40% lower; smaller and weaker wake zones	[18]
Witte et al.	Stereo-PIV + CFD + POD	3D whisker geometry significantly reduces lift/drag fluctuations; no Kármán vortices	Lift/drag fluctuation reduced by 90%; ~40% drag reduction at Re = 500	[55]
Kamat et al.	Finite element analysis	Geometric parameter λ/Dₘ strongly influences vibration suppression efficiency	Optimal VIV suppression when λ/Dₘ = 4.4–4.6	[38]

**Table 4:** Comparative effects of angle of attack (AOA) on vortex-induced vibration (VIV) suppression.

Researchers	Methodology	AOA Range (°)	Key Findings	Metrics/Conclusions	Ref.
Murphy et al.	Experimental comparison (wavy vs. smooth whiskers)	0, 90	Whisker geometry has minimal effect on frequency; AOA significantly affects vibration characteristics	Frequency highest and velocity lowest at AOA = 0°; inverse at AOA = 90°	[36]
Bunjevac et al.	PIV	0, 90	More stable wake and stronger VIV suppression at 0°; more turbulence at 90°	Vortex rapidly decays at AOA = 0°; intensified at AOA = 90°	[18]
Wang & Liu	PIV comparison (vs. elliptical/circular cylinders)	–30–30	Whisker structure shows minimal wake and weaker velocity fluctuations within ±30° AOA	Harbor seals can naturally maintain AOA within this range	[61]
Wang et al.	TR-PIV wind tunnel experiments	0, 30, 60, 90	Negligible vibration at AOA ≤ 30°; significant increase in amplitude at AOA > 30°	AOA significantly influences VIV response	[62]
Kim & Yoon	Experimental comparison (HSV vs. elliptical cylinder)	0–90	HSV shedding frequency increases then decreases with AOA; lower force coefficients than elliptical cylinder	HSV shows superior VIV suppression performance	[60]

**Table 5:** Comparative analysis of different methodological approaches used in VIV suppression studies.

Method Type	Advantages	Limitations
PIV experiments (e.g., Hanke [17], Bunjevac [18])	Enables visualization of real flow fields and acquisition of instantaneous velocity distributions	Limited spatial resolution; challenging to track complex unsteady flow structures over time
CFD simulations (e.g., Witte [55], Kamat [38])	Provides controlled parameters for evaluating structural variations on vortex dynamics	Requires assumptions of boundary conditions; difficult to account for environmental noise and stochastic disturbances
Scaled water tunnel experiments (e.g., Miersch [54])	Capable of replicating real-world flow perturbations and validating dynamic responses of biomimetic structures	Scale effects and material mismatches may reduce fidelity to actual biological structures
Comparative tests on biomimetic geometries (e.g., Song [56,59], Chen [58])	Highlights performance differences across multiple geometric configurations, emphasizing the advantage of biomimicry	Often relies on idealized numerical conditions or simplified models
FEA (e.g., Kamat [38])	Enables accurate modeling of structural mechanics, stress distribution, and fluid–structure interactions	Computationally intensive; complex to model; highly sensitive to material parameters and boundary conditions, which may not fully reflect real environments

**Table 6:** Performance comparison of other materials and seal whiskers.

Material	Young’s Modulus	Operational Lifetime in Water (Years)	Dynamic Bionic Compatibility
Natural Keratin (Seal)	≈2–4 GPa	25–30	Benchmark for bionic reference
PDMS	0.5–3 MPa	2–5	Soft–rigid balance, good dynamic match
Polyurethane	1–100 MPa	2–5	Flexible, good bionic response
Photopolymer Resin	1–3 GPa	<1	Moderately rigid, high-frequency performance
PEEK	≈3.5 GPa	5–10	Balanced stiffness, excellent durability

## Data Availability

The original contributions presented in this study are included in the article; further inquiries can be directed to the corresponding author.
